# Pre and postoperative psychological profile of children submitted to adenoidectomy and/or tonsillectomy

**DOI:** 10.1016/S1808-8694(15)31210-6

**Published:** 2015-10-20

**Authors:** Ilana Fukuchi, Meyre Maria Marques Morato, Rubens Ernani Cozeto Rodrigues, Giovana Moretti, Márcio Falcão Simone Júnior, Priscila Bogar Rapoport, Melissa Fukuchi

**Affiliations:** 1 Resident physician, Discipline of Otorhinolaryngology, Medical School ABC.; 2 Otorhinolaryngologist, SBORL. Assistant physician, Discipline of Otorhinolaryngology, Medical School ABC.; 3 Otorhinolaryngologist, SBORL. Assistant physician, Discipline of Otorhinolaryngology, Medical School ABC.; 4 Resident physician, Discipline of Otorhinolaryngology, Medical School, ABC.; 5 Resident physician, Discipline of Otorhinolaryngology, Medical School, ABC.; 6 Otorhinolaryngologist, SBORL. Ph.D. in Otorhinolaryngology, FMUSP. Faculty Professor, Discipline of Otorhinolaryngology, Medical School, ABC.; 7 Psychologist, Pontifícia Universidade Católica de Campinas.

**Keywords:** preoperative teaching, psychology, tonsillectomies, children

## Abstract

Adenoidectomy and/or tonsillectomy are the most frequent surgeries in otorhinolaryngology. Infantile psychological trauma may be caused by surgeries and anesthesia. **Aim:** To estimate the preoperative service offered to children and their responsible people by examining their psychological profile pre and postoperatively. **Study Design:** Clinical perspective. **Material and Method:** We have evaluated the medical chart of children between two and twelve years old who were submitted to adenoidectomy and/or tonsillectomy during February to December of 2003 and analyzed the psychological profile applied to the children and their responsible person. **Results:** Out of the total of 78 patients, 32 (41.0%) were in pre-school age and 46 (59.0%) in school age. The predominant feeling in pre-school age was fear (59.4%), while in school-aged children and their responsible guardian it was trust: 63.0% and 48.72%, respectively. As to expectation of surgery results, both children (73.08%) and their responsible people (96.15%) showed optimism. Introverted emotional temperament was observed in the majority of the children (52.56%) and their responsible people (51.28%). The emotional reaction at the immediate postoperative period of children and their guardians was calm: 68.18% and 97.73%, respectively. All children were psychologically apt to be submitted to the surgery. **Conclusion:** Independent of the predominant feeling or emotional temperament, good preoperative guidance is required. We have to offer preoperative teaching program that includes verbal descriptions of the procedures among the sensations to be experienced, allied with the interaction of children and parents, looking for reduction of anxiety, response to surgical stress and possible postoperative sequelae.

## INTRODUCTION

Adenoidectomy and tonsillectomy are historically the most frequently performed procedures in the ENT specialty, whose main incidence is in the pediatric population. Most of the children will have their first surgical intervention in Otorhinolaryngology ^1^.

More than 40 years ago, Eckenhoff documented that pediatric psychological traumas may result from surgery and anesthesia. Six-month-old to six-year-old children are the most susceptible to presenting post-hospitalization behavioral disorders owing to their limited capacity to deal with abstract thinking 2, 3.

Both surgeries in this study are considered elective surgeries, performed in a day hospital, that is, children come from the ambulatory, are operated on and discharged on the same day, except if there are complications. Given that they are scheduled surgeries, there is a preoperative preparation. According to Margolis and Gimberg, ideally there should be a preoperative educational program containing a verbal description of the procedure together with the sensations to be experimented, associated with interactions between parents and children ^3^.

Directed to expanding the physician-patient relationship, we assessed the preparation provided to children and responsible people in the preoperative period, analyzing the psychological profile pre and postoperatively. If we perform the appropriate preoperative preparation, we will reduce the level of anxiety and the response to surgical stress, possibly reducing postoperative sequels ^3^.

## MATERIAL AND METHOD

To carry out this study, we analyzed retrospectively the medical charts of children submitted to adenoidectomy and/or tonsillectomy at the Discipline of Otorhinolaryngology, Medical School ABC in the period between February and December 2003, performed at Hospital Estadual Mário Covas, in the city of Santo André - SP. We used the Brief Psychological Care Protocol used by the clinical psychology service of the department during the preoperative assessment of patients, comprising pre-anesthetic and psychological assessment.

In the psychological assessment, both the child and the guardian were interviewed by the psychologist and data were recorded in the protocol that also comprised patients’ data (complete name, age and gender), responsible person’s data (name, age, relation with the patient, profession, religion); type of surgery to be performed and scheduled data; predominant feelings of the patient and guardian, such as distress, anxiety, trust, fear (anesthesia, surgery, pain) and others; the expectations concerning the surgical outcomes and whether they were optimistic or concerned, emotional characteristic of both, whether they were introverted or extroverted, and finally psychological opinion and management.

A new psychological assessment was performed right after the surgery, as soon as the child returned to the room, comprising observation and investigation of feelings about the surgical outcomes both by the patient and the parents, if they were optimistic or concerned, and how was the patient when he/she came from surgery: crying, confused, complaining or calm. It was concluded by psychological opinion and management.

The patients were investigated concerning age, gender, type of surgery, predominant feeling, emotional temperament, and expectation about the surgical result and predominant feeling, emotional characteristic and expectation of surgical outcome of the accompanying person as well.

After the surgery, children and the responsible person were asked about their predominant emotional feeling when they arrived in the room.

## RESULTS

There were a total of 78 patients, 43 (55.1%) boys and 35 (44.9%) girls. Out of the total, 32 patients (41.0%) were pre-school children (2-6 years) and 46 patients (59.0%) were school children (7-12 years). The mean age was 6.62 ± 2.36 years.

None of the children had contraindications to the surgery according to the final psychological opinion. Only one patient was referred to therapy, but the surgery was not contraindicated.

**Graph 1:**
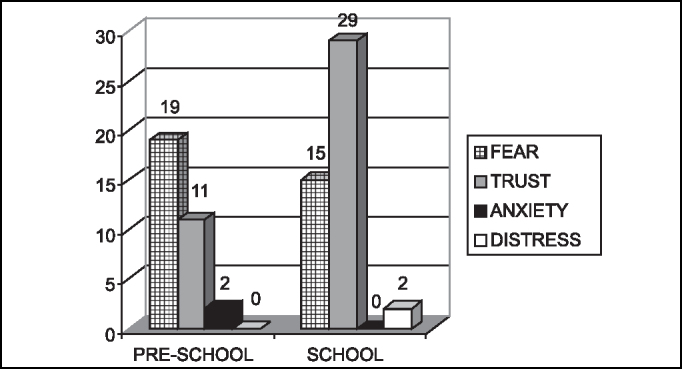
Predominant feeling in each child according to age range.

The predominant feeling in children according to age range can be seen in Graph 1.

**Graph 2:**
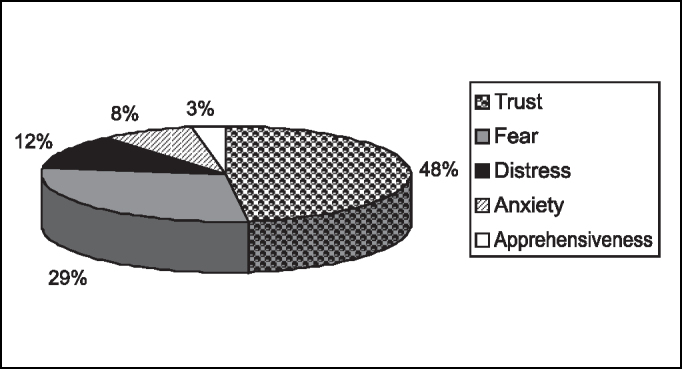
Predominant feeling in guardians.

The predominant feeling in responsible people can be seen in Graph 2. In those that there was predominance of fear, 12 (52.17%) reported the feeling because of anesthesia and 5 (21.74%) because of the surgery.

**Graph 3:**
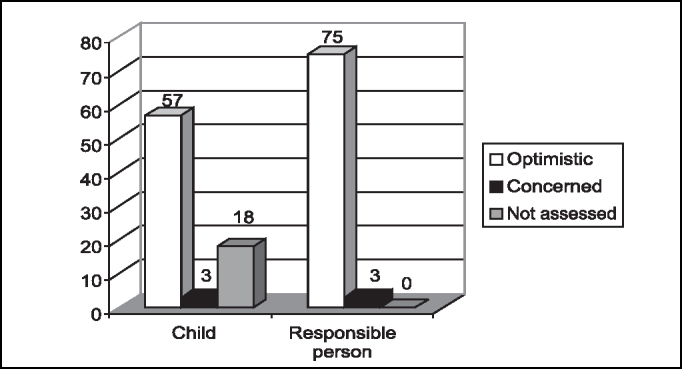
Expectation of surgical outcomes for children and responsible person.

The expectation of surgical outcomes for children and adults are shown in Graph 3. Out of the total number of cases that could not assessed, 88.89% (16 patients) were pre-school children.

**Graph 4:**
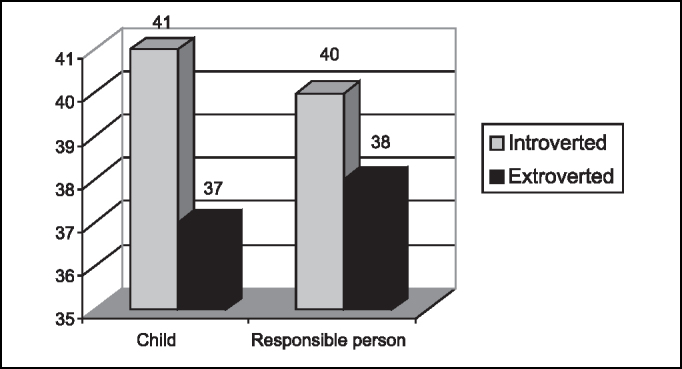
Emotional characteristic observed in children and in their responsible people.

Emotional characteristic observed in children and adults is shown in Graph 4.

**Graph 5:**
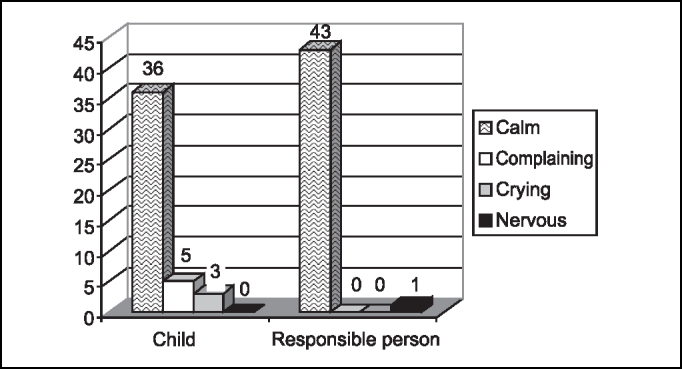
Emotional status of the child and the responsible person when they arrived to the room.

Emotional status of the child and responsible person right after the surgery is shown in Graph 5.

## DISCUSSION

In our Service of Otorhinolaryngology, all children with indication of adenoidectomy and/or tonsillectomy should go through an outpatient visit with the physician who is going to perform the surgery, a pre-anesthetic visit and psychological assessment. In the ENT visit, the child and the parents are instructed about pre and postoperative care, explanation about the surgery and type of anesthesia and which are the risks and benefits resulting from the procedure.

The preoperative information comprises fasting, medications that should not be taken and what time to arrive. Together with the Informed Consent Term about the surgical procedure, designed by the Brazilian Society of Otorhinolaryngology, we explain why to perform the surgery, early and late postoperative periods, possible complications and expected outcomes. We conclude by providing information on eating habits after the surgery, rest, drugs to be used (analgesics and antiemetics), return visit to the outpatient center and we provide our contact information in case of emergency or questions.

Pre-anesthetic assessment consists of a visit to the anesthesiologist in which preoperative tests are checked and a brief clinical history of the patient is made to identify some factor that might lead to risk in anesthesia.

The psychological assessment is made by one single psychologist, after the anesthesiologist has approved the patient, consisting of an interview with patient and family. In this session, data to fill out the brief form already described will be collected, classifying the patient as psychologically apt or not to undergo surgery.

To define the psychological profile of the subject it is important to characterize his/her personality and character.

It is by observing the reactions of subjects that the personalities are defined. To Lazarus, the personality is formed by relatively stable processes underlying to subjects’ reactions, in a dynamic organization of psychophysical systems (physical and psychological). The characteristic of temperament is the innate and biological basis of this process and the character values the personality, that is, it houses the social and cultural aspects that value the personality 4, 5.

To the Sao Paulo Society of Clinical Psychiatry, personality is a dynamic organization of traits inside you, formed by particular genes that we inherit, from the unique existence we have and the individual perceptions we have about the world, capable of making people unique in its way of acting and playing its social role. More important than identifying the personality of each subject is to determine their personality traits. They are persistent patterns in the way the reality is perceived, the person relates with oneself and the others and how she/she reasons. When personal traits are inflexible, rigid and poorly adapted to a harmonious life, leading to social and occupational damage or distress on people surrounding him or her, these personality traits are considered personality disorders. Personality disorder is a contraindication for an elective surgical procedure ^5^.

It is necessary to conceptualize the predominant feelings to understand whether there is any contraindication or not for the surgical procedure 7, 8, 9.

Distress and fear may be mistaken. Distress is defined by Karl Jaspers as a frequent and torturing feeling and that fear always refers to something, whereas distress does not have a focus object. Distress is a feeling whose onset takes place in widely configured, precise and determined physical structures, in which the knowledge processes that precede it are frequently more vague and undifferentiated. Fear means that someone fears something (surgery, anesthesia, pain, malpractice, injection) ^5^.

In general, anxiety is triggered by upset facts, suffering or loses. In other occasions there are concerns about money, health, safety. In anxiety, the trend is not to be concerned about oneself, leading to sloppy feeling and no concern about appearance. In general, there is concern about one topic rather than anything else. Reasoning gets difficult. There are problems of concentration, insomnia, and impatience plus memory disorder. The behavior is modified and depends on each ones’ personality, that is, it may be manifested by aggressive or depressive actions ^5^.

Distress, anxiety and fear may be minimized preoperatively by strengthening the physician-patient relationship at the moment in which they are talking about the surgery, risks and benefits of the procedure. It is mandatory and if there is failure to providing information, there may be difficult to repair postoperative behavior sequels^10^, ^11^.

According to Jung, there are two psychological temperaments: extroverted and introverted, but both are considered normal, because only exaggerated types would be considered pathological. The extroverted person is the one in which the psychological energy flows towards the object. The introverted, in turn, is the person in which the energy recesses in view of the object, because the object offers something threatening that affects the subject, but there is a moment of unconscious compensation that conveys energy to the object. Thus, the more introverted the emotional temperament, the more difficult it is to define a physician-patient relationship and the higher the risk the child will present post-hospital behavior modifications 6, 12.

Based on our study, we confirmed that only severe stages of distress, anxiety, fear and extroversion or introversion are capable of contraindicating a surgery. Regardless of the predominant feeling or emotional temperament of each child and their family members, preoperative preparation is required to minimize the sensations and to prevent their pathologic transformation 3, 13, 14. In our study, we had predominance of fear in pre-school children (59.4%) and of trust in school children (63%), and most of them were introverted, but there was no contraindication to the surgery. There was a pre-school children in whom fear was predominant, she was crying in the preoperative assessment and also in the postoperative visit, but the procedure was not contraindicated and this fact was explained by her young age - 2 years old, the youngest child in the sample.

The preoperative educational program proposed by Margolis in 1998 contains information about the surgical procedure and sensorial descriptive information, The author developed an interactive book in an easy non-medical language, colorful and directed to children who are going to undergo surgery, comprising figures (visual sensation), surgical instrument, surgical mask, oxymeter fixed in the finger (tactile sensation) and essences (inhaling gas), as olfaction sensation. In a study with 143 children divided into 2 groups (with and without access to the book), they demonstrated the impact of the book on children who were going to be operated on and their family members, associated with the changes in behavior seen in anesthetic induction and in the weeks after the surgery. In the group that had access to the book, there was increase in level of anxiety on the surgery day, but there was significant reduction of behavioral changes observed in the weeks following the procedures. 87% thought the book had helped their children and 83% said it helped themselves; 83% of the parents that had received the book were satisfied with the preoperative information they had learned as opposed to 66% in the group that had not read the book ^3^. In our study, we observed that 81.82% of the children were calm in the early postoperative period and they did not show any significant behavioral modifications.

In another study, Padfield reported that 73% of the patients aged between 3 and 9 years presented adverse behavioral disorders in the first 2 weeks after the surgery against 55% of the patients reported by Schmidt, who had played with medical equipment in the preoperative period. These studies, more than once, demonstrated the need to have a comprehensive preoperative educational program bringing patients and physicians together. Teaching what patients do not know or are not familiar with prevent psychological sequels or worsening of underlying conditions 7, 8, 15, 16.

## CONCLUSION

None of the children had negative psychological opinion that could contraindicate the surgery.

Based on our study, we confirmed that only severe stages of distress, anxiety, fear and extroversion or introversion are capable of contraindicating a surgery. Regardless of the predominant feeling or emotional temperament of each child and their family members, preoperative preparation is required to minimize the sensations and to prevent their pathologic transformation. When fear is the predominant feeling in adults, it refers to anesthesia and surgery, reinforcing the importance of appropriate preoperative preparation.

Thus, ideally, there should be a preoperative educational program that would contain a verbal description of the procedure together with the sensations experimented in it, associated with interaction of parents and children. Therefore, we should reduce the level of anxiety, the response to surgical stress, behavioral changes and possible postoperative sequels.
